# Does diastolic dysfunction precede systolic dysfunction in trastuzumab-induced cardiotoxicity? Assessment with multigated radionuclide angiography (MUGA)

**DOI:** 10.1007/s12350-015-0164-x

**Published:** 2015-06-06

**Authors:** E. J. Reuvekamp, B. F. Bulten, A. A. Nieuwenhuis, M. R. A. Meekes, A. F. J. de Haan, J. Tol, A. H. E. M. Maas, S. E. Elias-Smale, L. F. de Geus-Oei

**Affiliations:** Department of Radiology and Nuclear Medicine, Radboudumc, Nijmegen, The Netherlands; Biomedical Photonic Imaging Group, MIRA Institute for Biomedical Technology and Technical Medicine, University of Twente, PO Box 217, 7500 AE Enschede, The Netherlands; Department for Health Evidence, Radboudumc, Nijmegen, The Netherlands; Department of Medical Oncology, Radboudumc, Nijmegen, The Netherlands; Department of Cardiology, Radboudumc, Nijmegen, The Netherlands; Department of Radiology, Leiden University Medical Centre, Leiden, The Netherlands

**Keywords:** Breast cancer, monoclonal antibodies, cardiotoxicity, radionuclide imaging, diastolic function

## Abstract

**Background:**

Trastuzumab is successfully used for the treatment of HER2-positive breast cancer. Because of its association with cardiotoxicity, LVEF is monitored by MUGA, though this is a relatively late measure of cardiac function. Diastolic dysfunction (DD) is believed to be an early predictor of cardiac impairment. We evaluate the merit of MUGA-derived diastolic function parameters in the early detection of trastuzumab-induced cardiotoxicity (TIC).

**Methods and Results:**

77 trastuzumab-treated patients with normal baseline systolic and diastolic function were retrospectively selected (n = 77). All serial MUGA examinations were re-analyzed for systolic and diastolic function parameters. 36 patients (47%) developed SD and 45 patients (58%) DD during treatment. Both systolic and diastolic parameters significantly decreased. Of the patients with SD, 24 (67%) also developed DD. DD developed prior to systolic impairment in 54% of cases, in 42% vice versa, while time to occurrence did not differ significantly (*P* = .52). This also applied to the subgroup of advanced stage breast cancer patients (*P* = .1).

**Conclusions:**

Trastzumab-induced SD and DD can be detected by MUGA. An impairment of MUGA-derived diastolic parameters does not occur prior to SD and therefore cannot be used as earlier predictors of TIC.

## Introduction

Trastuzumab is a recombinant humanized monoclonal antibody successfully used for the treatment of human epidermal growth factor receptor2(HER2)-positive breast cancer. It prolongs the survival in metastatic disease and, when combined with adjuvant chemotherapy, also improves outcome in early stage breast cancer.^1^ However, its use is associated with an increased risk of cardiotoxicity, which frequently leads to temporary or definite discontinuation of trastuzumab therapy.

The overall incidence of trastuzumab-induced cardiotoxicity (TIC) varies according to the definition used, but ranges from 2% to 7% for trastuzumab monotherapy, 2% to 13% for trastuzumab combined with paclitaxel, and can be as high as 27% when used in combination with anthracyclines.^2^ TIC is often transient after discontinuation of trastuzumab, but may be irreversible if detected in a late stage.^3^,^4^ Cardiotoxicity may be symptomatic with signs and symptoms of congestive heart failure, but in the vast majority, it is asymptomatic. If treatment with trastuzumab is continued despite a decrease in cardiac function, severe and even irreversible loss of cardiac function may occur. Thus, close cardiac monitoring in patients undergoing trastuzumab therapy is of paramount importance.

Evaluation of the left ventricular systolic function through measurement of the left ventricular ejection fraction (LVEF), using multigated radionuclide angiography (MUGA) or echocardiography, is the accepted method in assessing cardiac function during trastuzumab treatment. However, deterioration of LVEF only becomes apparent if the myocardium has lost its considerable functional reserve, and therefore is a relatively late expression of left ventricular dysfunction.^5^ The detection of changes in cardiac parameters preceding decrease in LVEF allows the identification of patients who might benefit from early cardioprotective measures. Consequently, the likelihood of discontinuation of effective systemic treatment, potentially compromising the patients’ long-term outcome, might be markedly reduced.

It has been shown that diastolic impairment of the left ventricle occurs before deterioration in LVEF in anthracycline-induced cardiotoxicity (AIC).^6^,^7^ For TIC, Lange et al and Dores et al echocardiographically studied diastolic dysfunction (DD) in relatively small groups, but their results contradict.^8^,^9^ Cochet et al evaluated MUGA-derived diastolic parameters and found no significant impairment of diastolic function during trastuzumab therapy, but they included only early stage breast cancer patients, used a short follow-up period of 1 year and did not study the time interval of the first impairment of diastolic function.^5^

MUGA remains a reference technique for the evaluation of LVEF because of its standardized and reproducible outcome. Apart from routine use for LVEF determination, a MUGA study can provide diastolic parameters. Peak filling rate (PFR) and time to peak filling rate (TPFR) derived from the LV time-activity curve are the commonly used diastolic parameters. A decrease and/or delay in these parameters are typical disturbances of a normal filling pattern. The lower limit of PFR is 2.50 end-diastolic volumes per second (EDV/s). The TPFR is expected to be less than 180 ms in a normal subject.^10^,^11^ Studies have demonstrated that diagnosing diastolic abnormalities by a low PFR on MUGA correlates well with changes seen on Doppler echocardiography.^12^

We hypothesized that a change in diastolic function parameters measured by MUGA precedes deterioration in LVEF. Therefore, we evaluated MUGA-derived diastolic and systolic function parameters in breast cancer patients treated with trastuzumab, in order to determine whether diastolic function parameters can be used as early detectors of TIC.

## Methods

### Patient Population

HER2-positive female breast cancer patients, treated with trastuzumab from 2003 to 2011 in the Radboud University Medical Centre Nijmegen and with a MUGA examination before trastuzumab, were retrospectively selected from registered data (n = 105). Both women with metastatic disease and early stage breast cancer were included. Patients with a prior history of severe cardiac events or an LVEF < 50% were excluded from trastuzumab therapy (n = 1). Patients with abnormal baseline PFR or TPFR values (n = 27) were excluded from our primary analysis, but evaluated in a separate subgroup analysis. The remaining study population consisted of 77 subjects. Of these patients, the history of MUGA scans and cardiac disease (until January 2015) was collected and analyzed.

### Treatment Regimens

After surgical staging, patients were selected for loco-regional therapy and adjuvant treatment. In the non-metastatic setting, patients received adjuvant chemotherapy with doxorubicin, cyclophosphamide, a taxane, and trastuzumab. In the metastatic setting, patients received several chemotherapeutic regimens, consisting of anthracyclines, taxanes, bevacizumab, capecitabine, CMF (cyclophosphamide, methotrexate, fluorouracil), and FEC (fluorouracil, epirubucin, cyclophosphamide) in divergent order, but all patients received trastuzumab during a certain time span. Trastuzumab was administered in a standard regimen: first dose at 8 mg/kg followed by a 3-week schedule of 6 mg/kg diluted in 250 mL of sodium chloride 0.9%. When SD was observed, trastuzumab treatment was ceased or postponed until LVEF was recovered.

### Multigated Radionuclide Angiography

All subjects underwent cardiac monitoring by serial MUGA examinations at our institution. The baseline MUGA was obtained before the start of treatment, with an every 3-month assessment during therapy. The final examination was acquired 1 year after completion of trastuzumab or earlier in cases where discontinuation of therapy was indicated. MUGA examinations were performed in supine position, with multiple gated ECG-triggered sampling after injection of 740 MBq (20 mCi) 99m-technetium radio-labeled red blood cells and with acquisition from the left anterior oblique (LAO) view. 32 frames per cardiac cycle were used. The R-R interval and the heart rate (beats/min) were recorded. Cardiac cycles with an R-R interval outside 20% of the median were rejected. Acquisition lasted till 600 accepted cycles were included.

MUGA recordings were re-analyzed for systolic and diastolic function parameters. The reference parameter for systolic function was LVEF, while reference parameters for diastolic left ventricular function were PFR and TPFR. LVEF was calculated from the time-activity curve; PFR and TPFR were acquired from the first derivation of the time-activity curve after cycle-dependent background correction, by a clinically valid software program (Hermes Medical Solutions, Stockholm). Cardiac systolic dysfunction (SD) was defined as a LVEF < 50% or a drop of 10 or more absolute points in baseline LVEF.^13^ DD was defined as a PFR < 2.5 EDV/s or a TPFR > 180 ms.^5^,^11^

### Statistical Analysis

For all individuals, a time registration (in days) was made from baseline MUGA up to the last examination after treatment. Time intervals to first SD or DD were calculated. If DD is an early detector of TIC, the time interval to first impairment of diastolic parameters should be smaller than the time interval to first impairment of systolic parameters. To test these intervals, the following convention was used: if no impairment of systolic parameters was seen, the time to SD was set to the follow-up time. The same was done if no DD was observed. A comparison of time intervals of the two parameters was made using the Wilcoxon signed-rank test.

In-time differences of LVEF, PFR, and TPFR were tested for significance with the paired *t* test and a Holms correction for multiple testing was done.^14^ For the in-time assessment of these parameters, the first six MUGA examinations were used. The level of significance was set at 0.05. All calculations were made with SPSS 20.0 for Windows.

## Results

Demographic and clinical characteristics of the study population are displayed in Table 1. The number of MUGA scans performed ranged from two to nineteen per patient (median six, n = 46). The number varied with disease stage and the patient’s tolerance of trastuzumab (i.e., decrease of LVEF). In patients with early stage breast cancer, trastuzumab was administered for 1 year, corresponding with 17 cycles of 21 days. Generally, patients with metastatic disease continued trastuzumab therapy longer and underwent more MUGA examinations. Those with poor tolerance of trastuzumab and the possible need for therapy withdrawal were monitored more frequently, with an increasing number of scans and shorter time intervals. Necessity of additional scans was based on clinical grounds and at the physician’s discretion. In the vast majority, MUGA examinations were performed in a 3 monthly interval during therapy. Follow-up of symptoms indicating cardiac disease ranged from 33 to 117 months. One patient developed clinical symptoms of cardiac failure during therapy and follow-up. This patient showed both DD and SD on MUGA, respectively, after 62 and 129 days. None of the other patients received additional cardiac intervention during trastuzumab treatment or follow-up.

**Table 1:** Clinical characteristics of study population with normal (n = 77) and abnormal (n = 27) diastolic function at baseline

	Normal diastolic function		Abnormal diastolic function		Significance (*P*)
	N (%)	Mean (SD)	N (%)	Mean (SD)	
Clinical data					
Age (years)		50.3 (10.4)		55.4 (7.8)	<.05
BMI (kg/m^2^)		25.2 (4.4)		27.8 (5.2)	<.05
Cardiovascular risk factors					
Hypertension	11 (14)		10 (37)		
Diabetes	3 (4)		1 (4)		
Smoking	12 (16)		6 (22)		
Use of AT blockers	3 (4)		5 (19)		
Use of beta blockers	7 (9)		11 (41)		
Cancer characteristics					
Early stage breast cancer	64 (83)		16 (59)		
Metastatic breast cancer	13 (17)		11 (41)		
Location					
Right breast	31 (40)		11 (41)		
Left breast	44 (57)		13 (48)		
Both sides	2 (3)		3 (11)		
History of (neo)adjuvant anthracyclines	63 (82)		20 (74)		
Left side radiation therapy	27 (35)		7 (26)		
MUGA data (baseline)					
General					
Heart rate (beat/min)		76 (10.7)		76 (14)	NS
R-R interval (ms)		801 (104)		820 (161)	NS
Systolic function					
LVEF (%)		63.6 (6.6)		59.9 (6.0)	<.05
PER (EDV/s)		3.55 (0.59)		3.32 (0.52)	NS
TPER (ms)		133 (20)		145 (25)	<.05
Diastolic function					
PFR (EDV/s)		3.42 (0.74)		2.74 (0.66)	<.01
TPFR (ms)		137 (17)		190 (44)	<.01

*BMI*, Body mass index; *AT,* angiotensin; *MUGA*, multigated radionuclide angiography; *LVEF*, left ventricular ejection fraction; *PER*, peak ejection rate; *EDV*, end-diastolic volume; *TPER*, time to peak ejection rate; *PFR*, peak filling rate; *TPFR*, time to peak filling rate; *NS*, not significant

**Figure 1:**
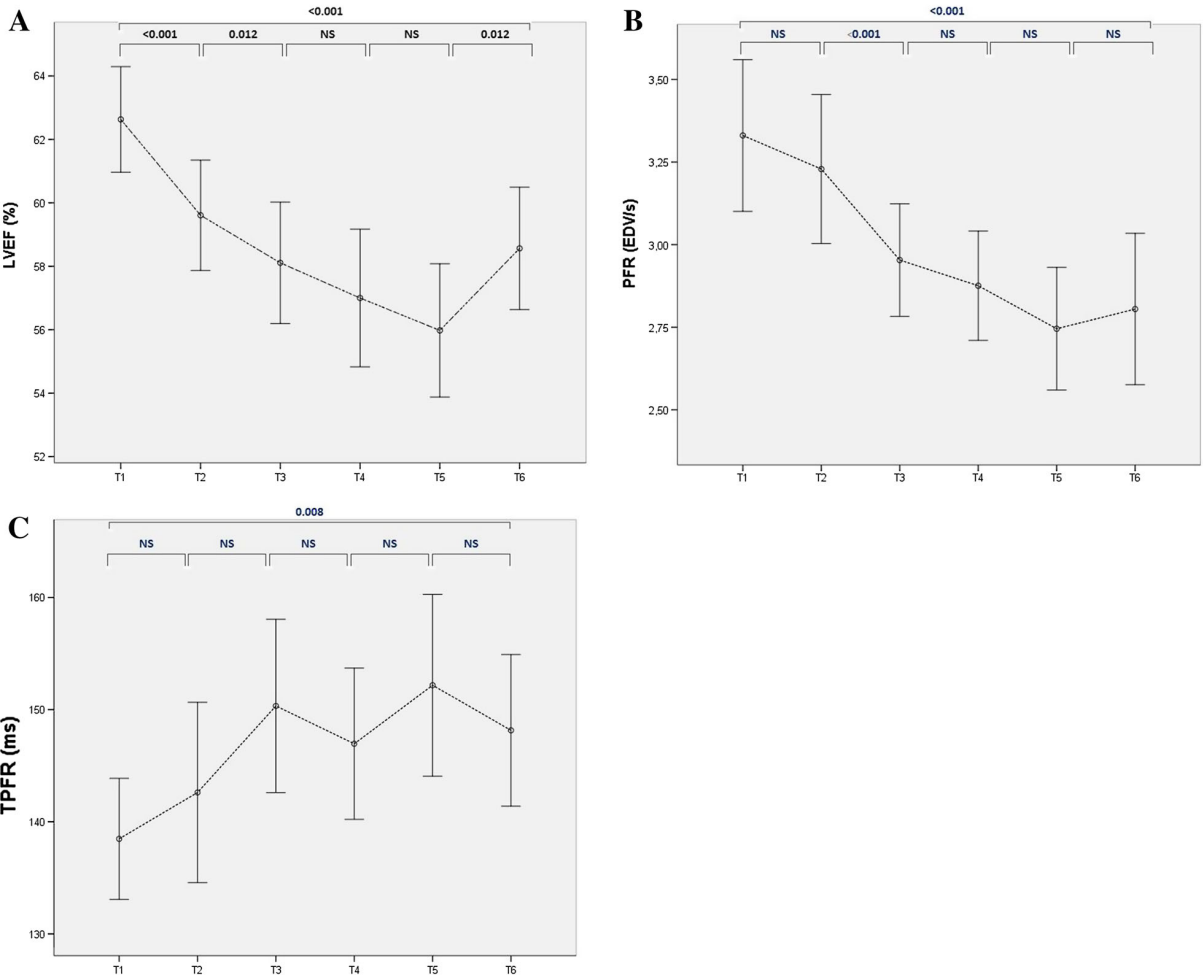
Serial results of left ventricle ejection fraction (LVEF, A), peak filling rate (PFR, B) and time to peak filling rate (TPFR, C) at six time points, T1 depicting baseline scan and T2-T6 consecutive MUGA scans. Number of evaluated MUGA scans: T1 = 77, T2 = 74, T3 = 72, T4 = 63, T5 = 46, and T6 = 46. In general, MUGA examinations are acquired every 3 months, but can be made earlier at physicians’ discretion. Individual time points could therefore deviate from population mean. All values are mean with 95% confidence interval

Of the 77 subjects included in our study, SD at any point in time during or after treatment was detected in 36 patients (47%, Table 2). Mean LVEF significantly decreased in time (Figure 1), with a mean LVEF of 58.6% at MUGA 6. Of these 36, 11 (31%) still showed SD at the last MUGA. 45 patients (58%) showed DD at any time point, of which 39 patients showed a PFR of <2.5 EDV/s and 26 showed a TPFR > 180 ms. Nineteen patients had both abnormal PFR and TPFR. Both parameters significantly decreased in time (Figure 1), with a mean PFR of 2.8 EDV/s and a mean TPFR of 148 ms at MUGA 6. DD was present in 29 of 45 patients (64%) at the last MUGA.

**Table 2:** Frequencies of SD and DD in the total population (n = 77)

	N (%)
SD	36 (47)
DD	45 (58)
PFR < 2.5 EDV/s	39 (87)
TPFR > 180 ms	26 (58)
Abnormal PFR + TPFR	19 (25)
DD + SD	24 (31)
DD before SD	13 (54)
DD after or concurrent with SD	11 (46)

*SD*, Systolic dysfunction: LVEF < 50% or a drop of 10 or more absolute points in baseline LVEF; *DD*, diastolic dysfunction: PFR < 2.5 EDV/s or TPFR > 180 ms; *PFR* peak filling rate; *TPFR*, time to peak filling rate

Of the 36 patients with LVEF values below normal, 24 (67%) showed also deterioration of PFR or TPFR. Of these 24 patients, who were considered to have both SD and DD, 13 patients (54%) showed DD prior to SD and 10 patients (42%) vice versa. Five of thirteen patients recovered to baseline systolic function. In one patient, a simultaneous decrease in systolic and diastolic function was observed. Furthermore, in 21 of 77 patients (27%), DD occurred without SD, while SD without DD occurred in 12 patients (16%).

**Figure 2:**
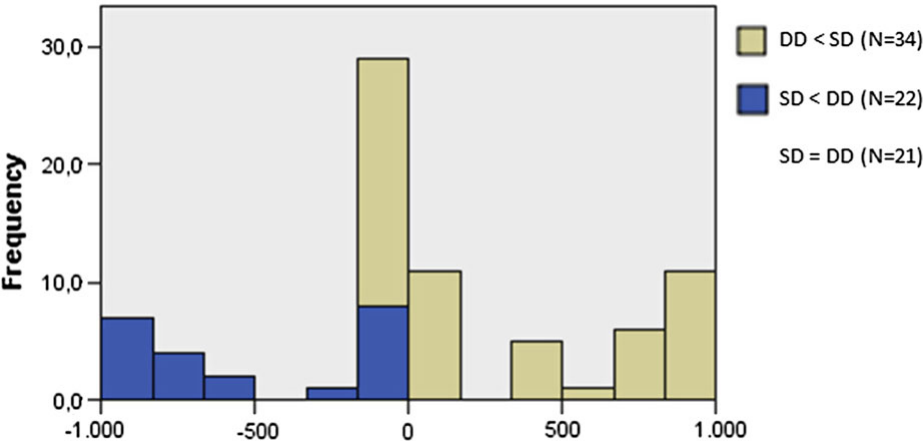
Visualization of Wilcoxon signed-rank test showing DD before SD in 34 cases and SD before DD in 22 cases. In the other 21 cases, both events were simultaneous or did not even occur. There was no significant difference in both groups (*P* = .09)

In the subgroup of 24 patients, the median time to the first decrease was 122 days (range 36-581) for SD and 121 days (range 9-672) for DD, which was not significantly different (Wilcoxon signed-rank test, *P* = .56). Comparison of time intervals between the first moment of SD and DD in all patients, using the Wilcoxon signed-rank test, did not show any significant difference in both parameters (*P* = .10, Figure 2).

### Subgroup Analyses

Next, we performed a subgroup analysis of patients with early stage and advanced stage (i.e., metastatic) breast cancer, since the latter group may have received trastuzumab over a longer period of time, whereas patients with early breast cancer were treated during a fixed period of 12 months (Table 3). In both patient groups, there was no significant difference detected.

**Table 3:** SD and DD in patient subgroups

	Breast cancer stage		Anthracycline use		Left-sided radiation therapy	
	Early (N = 64)	Advanced (N = 13)	Naive (N = 14)	Treated (N = 63)	Naive (N = 50)	Treated (N = 27)
SD						
N (%)	29 (45)	7 (54)	6 (43)	30 (48)	21 (42)	15 (56)
Median, days (range)	129 (36–581)	138 (49–407)	150 (49–407)	122 (36–581)	133 (36–538)	129 (62–581)
DD						
N (%)	34 (53)	11 (85)	12 (86)	33 (52)	28 (56)	17 (63)
Median, days (range)	149 (17–672)	136 (9–573)	111 (9–573)	157 (18–672)	111 (18–575)	156 (9–663)
Wilcoxon signed-rank test (SD vs DD), *P*	0.26	0.12	0.02*	0.51	0.15	0.39

*SD*, Systolic dysfunction: LVEF < 50% or a drop of 10 or more absolute points in baseline LVEF; *DD*, diastolic dysfunction: PFR < 2.5 EDV/s or TPFR > 180 ms; *PFR*, peak filling rate; *TPFR*, time to peak filling rate

* *P *< 0.05

To evaluate anthracycline influence, another subgroup analysis was done in the small subgroup of anthracycline naïve patients (n = 14, Table 3). In this group, we detected a significant difference in time to DD compared to SD (*P* = .02).

Additionally, to assess radiation therapy influence, a subgroup analysis was performed in left-sided radiotherapy naïve patients (n = 50). Although DD developed before SD (median time 111 vs 133 days), this was not significant according to the Wilcoxon signed-rank test (*P* = .15, Table 3).

### Subgroup Analysis of Patients with Baseline DD

Twenty-seven patients were excluded from the primary analysis due to DD at baseline. Of these, 10 patients had a PFR < 2.5 EDV/s, twelve a TPFR > 180 ms, and five both. Patient characteristics are displayed in Table 1. Besides a significant difference in diastolic parameters, age, BMI, LVEF, and PER differed from the initial patient group (Table 1).

Seventeen patients (63%) developed SD in a median time of 116 days (range 28-1206). Mean LVEF, however, did not significantly change in the course of trastuzumab use (*P* = .08). Also, diastolic function remained constant (PFR *P* = .14, TPFR *P* = .75).

## Discussion

Since subclinical signs of cardiac dysfunction are supposed to predict the development of future heart failure, many parameters of diastolic and systolic function have been proposed to detect early cardiotoxicity.^15^ However, conclusive evidence on the use of the most optimal and meaningful combination of cardiac function parameters is still lacking.

The present study retrospectively investigated whether impairment of systolic function is preceded by MUGA-detectable DD in a group of 77 female breast cancer patients undergoing trastuzumab therapy. The results of this study showed a nearly even number of patients with DD preceding SD (54%), as compared to the number of patients with the opposite order (42%). However, in 27% of patients, DD occurred without SD, while in 16% SD was observed without deterioration of diastolic function. Furthermore, in the relatively small subgroup of advanced breast cancer patients, up to 85% showed a decrease in diastolic function, in contrast with 54% SD. The time interval in which the patients developed either one did not differ significantly. The rates of DD and SD in the small subgroup of anthracycline naïve patients were 86% and 43%, respectively, with significantly different time intervals. For radiation therapy naïve patients the differences were small (SD 42% and DD 56%). In general, both LVEF and diastolic parameters significantly decreased due to trastuzumab admission (Figure 1). Lastly, in the subgroup of patients that suffered from baseline DD, 63% also developed SD, but there was no significant decrease in systolic or diastolic function.

Diastolic impairment is presumed to be an early predictor of developing heart failure, thought to develop before systolic impairment in AIC,^5^,^6^,^16^,^17^ while this is not yet studied in TIC. In AIC, the endocardium is most susceptible to the deleterious effects of anthracyclines, resulting in interstitial fibrosis, while in the same stage of disease the mid-myocardial fiber layers are not yet affected.^18^ In TIC, the main pathophysiologic mechanism is inhibition of HER2 in cardiomyocyte tissue. The HER2 pathway is required for cell survival and continuing function and seems to be stimulated in situations of myocardial stress, such as anthracycline treatment.^19^ HER2 inhibition results in depletion of ATP and subsequent dysfunction of contractility.^20^ For TIC, in contrast to AIC, it has been shown that it is not dose dependent, is often transient after drug discontinuation, and can be safely re-administered after recovery of left ventricular ejection fraction.^19^

The findings of the present study are supported by the results of Lange et al^8^ and Dores et al^9^ and are also in agreement with a previous report on short-term anthracycline-based chemotherapy, showing simultaneous impairment of left ventricular systolic and diastolic radionuclide parameters.^21^ Interestingly, although developed simultaneously, in our population we found that 31% and 64% of patients showed persistent SD and DD at their last MUGA scan. However, only one patient developed symptomatic heart failure. This patient showed persistent DD, but recovered systolic function on the last MUGA.

Reduced diastolic (time to) peak filling rates are thought to detect early chemotherapy-induced cardiotoxicity with greater sensitivity, but to date the additional value of these parameters in the prediction of future heart failure, compared to serial MUGA-based assessment of LVEF, has not been proven.^22^ Our study evaluated the same parameters in a trastuzumab-treated patient population and resulted in comparable findings. It is the first study, however, that evaluates the time to occurrence and includes patients with metastatic disease in the evaluation.

In general, nearly all patients that do not have metastases at diagnosis are treated with anthracyclines. Consequently, it is expected that subgroups of anthracycline naïve and advanced stage patients are generally the same and display a (nearly) identical cardiac dysfunction pattern. Interestingly, we found a significant shorter time interval to DD in the anthracycline naïve subgroup, while subgroup analyses of early vs advanced (i.e., with metastases) stage did not show such a difference. However, the number of patients in both subgroup analyses is small and therefore no conclusions can be made based on this observation.

In general, reproducibility is very important for any imaging method. In a recent review, the inter- and intra-observer reproducibility of MUGA-derived LVEF turned out to be near perfect.^23^ The reproducibility of PFR and TPFR was already known to be excellent.^24^

Several other modalities in the early detection of cardiotoxicity have been studied, although they mainly focus on AIC. The two most promising cardiac serum biomarkers are (high-sensitivity) Troponin I (TnI) and N-terminal brain natriuretic peptide (NT-proBNP). A study in 251 women receiving trastuzumab identified TnI as a predictor of cardiotoxicity risk, but all patients had a history of anthracycline treatment. It is therefore not known whether increased TnI reflected AIC catalyzed by trastuzumab, or true TIC.^21^

Other imaging modalities such as echocardiographic strain (rate) imaging are thought to track early changes in cardiac function by measuring the relative deformation of myocardial segments and describing the contraction/relaxation pattern of the heart.^22^,^25^ Although only few studies assess the use of echocardiographic strain (rate) imaging in TIC, these suggest that several strain-derived parameters can be used as independent early predictors for TIC.^26^-^28^

^123^I-*meta-*iodobenzylguanidine (^123^I-*m*IBG) is an ^123^I-labeled norepinephrine analog, used to noninvasively assess the sympathetic myocardial innervation. Because ^123^I-*m*IBG is not metabolized, it deflects neuronal integrity and eventually cardiac status.^29^ To date, there is no experience in the use of ^123^I-*m*IBG for the evaluation of TIC, except for a small study in 9 patients with a confirmed LVEF decrease, that suggests that risk stratification and monitoring in this group is possible.^30^

Cardiac magnetic resonance imaging (CMR) has been studied in the assessment of TIC. The main finding in TIC is delayed enhancement of the LV lateral wall in the mid-myocardial segment.^27^,^31^ In AIC, T1-weighted hyperenhancement within three days of the first administration is associated with reduced LVEF on day 28 after chemotherapy. Threshold values predicting LVEF impairment are not yet known. Although CMR is expensive, time-consuming, and less widely available, its great accuracy and reproducibility will render CMR an important cardiotoxicity imaging modality in the near future.^20^

### Study Limitations

In our study group, prior treatment with anthracyclines, radiotherapy, and/or concomitant heart failure therapy (angiotensin blockers and beta blockers) could have influenced the overall incidence of SD and DD. This, however, adequately reflects the clinical situation and gives the study a higher relevancy and applicability. Above that, our aim was to detect a difference in the time interval to SD or DD, not to estimate the overall incidence of cardiac dysfunction. All the primary study subjects had normal systolic and diastolic baseline values and heart failure therapy was hardly used at baseline (Table 1).

We did not observe a statistical significant difference in time to SD and DD between early and advanced stage breast cancer patients or between patients that were treated with or naïve to left-sided radiation therapy. This could be due to the small size of the subgroups. However, we did observe a significant difference in patients treated with or naïve to anthracyclines, which is expected to be even more significant if more patients had been included. Therefore, we think this reflects a relevant finding of our subgroup analysis.

In our institution, cardiotoxicity is defined as an absolute drop of 10 percent points in LVEF or a decrease below 50% in patients with baseline LVEF > 50%, thereby reducing the chance of false-positive test results. However, more recent and commonly used guidelines define cardiotoxicity as a reduction of LVEF of ≥5% to <55% with symptoms of heart failure or an asymptomatic reduction of the LVEF of ≥10% to <55%.^32^ Rates of SD may differ according to the definition used, impeding inter-study comparison. The definition used in our study may have led to an underestimation of SD in our population, however, without affecting study outcome.

Despite this, we found a high rate of SD in our study population (47%), as well as an even higher rate of DD (58%), which is discordant with reported literature figures.^2^ There are several possible explanations for this. First, this could be due to the fact that patients with metastatic disease, with a generally longer duration of treatment and thereby longer exposure to the deleterious effects on cardiac function, were also included in our study. Especially the rate of DD in the advanced stage breast cancer patient group was markedly higher than in the early stage breast cancer group (85% vs 53%). However, rates of SD in both groups were only slightly different: 45% in the group with early stage breast cancer, and 54% in the group with metastatic disease. Another explanation for the high incidence of SD could be that due to the retrospective and clinical nature of the study, patients with suboptimal cardiac function are still included for trastuzumab treatment, while these patients might have been excluded in a prospective trial. Consequently, our group reflects a realistic group of breast cancer survivors and is not biased toward optimal cardiac function. Finally, in our institution, MUGA examinations were done in a 3-month fashion, while in other institutions this period might be longer and minor changes in cardiac function are not detected.

## Conclusion

The results of the present study show that both trastzumab-induced SD and DD can be detected by MUGA, but that there is no certain order of occurrence. Therefore, MUGA-derived diastolic parameters seem not suitable as earlier predictors of TIC. Newer promising imaging strategies in the early screening for potential cardiotoxicity have been proposed. Further clarification about the predictive value of these techniques is much awaited.

## New Knowledge Gained

There is no difference in time interval to MUGA-derived SD and DD in a homogenous group of trastuzumab-treated breast cancer patients.

MUGA-derived DD seems not suitable for early detection of TIC.

## Abbreviations

SD: Systolic dysfunction
DD: Diastolic dysfunction
AIC: Anthracycline-induced cardiotoxicity
TIC: Trastuzumab-induced cardiotoxicity
LVEF: Left ventricle ejection fraction
MUGA: Multigated radionuclide angiography
PFR: Peak filling rate
TPFR: Time to peak filling rate
EDV: End-diastolic volume
HER2: Human epidermal growth factor receptor 2
